# Impact of Weight Loss on Testosterone Levels: A Review of BMI and Testosterone

**DOI:** 10.7759/cureus.76139

**Published:** 2024-12-21

**Authors:** Okelue E Okobi, Paola Khoury, Raul J De la Vega, Raphael S Figueroa, Dhaarak Desai, Bernadin Dina A Mangiliman, Olga L Vera Colon, Rodolfo J Urruela-Barrios, Abdul K Abdussalam, Miguel Diaz-Miret, Sergio Hernandez Borges

**Affiliations:** 1 Family Medicine, Medficient Health Systems, Laurel, USA; 2 Family Medicine, Lakeside Medical Center, Belle Glade, USA; 3 Family Medicine, Larkin Community Hospital Palm Springs Campus, Miami, USA; 4 Family Medicine, Larkin Community Hospital Palm Springs Campus, Hialeah, USA; 5 Family Medicine and Community Medicine, Larkin Community Hospital Palm Springs Campus, Hialeah, USA; 6 Internal Medicine and Family Medicine, Larkin Community Hospital Palm Springs Campus, Miami, USA

**Keywords:** bariatric surgery, body mass index (bmi), hormonal health, obesity, testosterone levels, weight loss

## Abstract

The purpose of this review is to explore the relationship between weight loss (WL), specifically reductions in body mass index (BMI), and increases in testosterone levels. Obesity and excess body fat are linked to reduced testosterone levels, which can lead to metabolic dysfunctions, reduced libido, and diminished muscle mass. To attain this purpose, this review will summarize current evidence on how weight reduction interventions, including dietary changes, exercise, and bariatric surgery, affect testosterone production in overweight and obese individuals. WL, particularly through fat reduction, has a positive influence on testosterone levels. Both moderate and significant reductions in BMI are associated with notable increases in serum testosterone levels. Dietary interventions, particularly low-carbohydrate and Mediterranean diets, have been linked to increased testosterone production in men with obesity. Exercise, particularly resistance training, has also been shown to improve hormonal profiles by lowering fat mass and boosting testosterone levels. Additionally, bariatric surgery has been identified as one of the most effective methods for increasing testosterone in morbidly obese individuals, with improvements sustained over time. The findings have indicated that there is strong evidence that WL, particularly through reductions in BMI, leads to increased testosterone levels. This relationship is mediated by improvements in insulin sensitivity, reduced inflammation, and lower levels of aromatase activity (the enzyme that converts testosterone to estrogen in fat tissue). Effective interventions, including diet, exercise, and bariatric surgery, have the potential to restore hormonal balance, improving overall health outcomes for men with obesity or higher BMI. Further research is needed to optimize interventions and explore long-term benefits.

## Introduction and background

Testosterone, the primary male sex hormone, is crucial in regulating reproductive health, muscle mass, fat distribution, and overall metabolic function. The balance of this hormone is sensitive to various factors, including body weight and composition. As obesity rates rise globally, research has increasingly focused on the relationship between body mass index (BMI) and testosterone levels, especially in the context of how weight loss (WL) can influence hormonal health [1-3]. Obesity is often associated with hormonal imbalances, particularly in men, where it is linked to lower levels of testosterone, a condition known as obesity-induced hypogonadism. The effects of excess adiposity extend beyond reproductive health, potentially impacting metabolic function, cardiovascular risk, and quality of life [1-5].

Obesity-induced hypogonadism is a significant public health issue, particularly among men. As of 2023, more than one in three adults (35%) in 23 states are classified as having obesity. Prior to 2013, no state reported an adult obesity prevalence of 35% or higher. Presently, at least one in five adults (20%) in every U.S. state is living with obesity [3-6]. Approximately 42% of adults in the U.S. are classified as obese, with up to 40% of obese men experiencing hypogonadal symptoms [6-8]. The prevalence of testosterone deficiency in this group ranges from 20% to 50%, depending on the severity of obesity and associated metabolic conditions [6-9].

Obesity is a complex disorder characterized by excessive fat accumulation resulting from an imbalance between caloric intake and energy expenditure. Obesity-induced hypogonadism is a complex endocrine disorder primarily driven by excessive adipose tissue, which acts as an endocrine organ secreting hormones and inflammatory mediators [1-4,6-8]. Increased adiposity leads to elevated levels of adipokines, such as leptin, causing disruptions in the hypothalamic-pituitary-gonadal (HPG) axis. This condition often results in insulin resistance, characterized by hyperinsulinemia that reduces sex hormone-binding globulin (SHBG) levels, affecting testosterone availability [7-9]. Furthermore, higher aromatase activity in adipose tissue converts testosterone to estradiol, further suppressing the HPG axis. Chronic low-grade inflammation and oxidative stress associated with obesity further impair Leydig cell function in the testes, contributing to decreased testosterone synthesis [6-9]. Alterations in gonadotropin levels, particularly reduced luteinizing hormone (LH), compound this hormonal imbalance. The resulting low testosterone levels create a vicious cycle that exacerbates metabolic dysfunction, increasing the risk of comorbid conditions like cardiovascular disease and type 2 diabetes [9-16].

Generally, maintaining a healthy weight is vital for overall well-being, and the bidirectional correlation between WL and hormonal changes has become a subject of immense interest within the scientific and research communities. A key aspect of the bidirectional correlation is the impact of WL on testosterone levels, an important hormone in men’s health and functioning. Testosterone hormone plays a significant role in body composition regulation, and reduction in its levels has been linked to an increment in body fats and consequent decrement in lean muscle mass [4-6,10-14]. On the other hand, obesity has been associated with decrements in testosterone levels, possibly as a result of aspects like alteration of the levels of SHBG and insulin resistance [4-8]. Nevertheless, the bi-directional correlation between WL and testosterone levels is intricate and has not been fully understood. Several recent studies have made efforts to offer insights into the complex relationship. In overweight and obese individuals, WL has been found to impact testosterone levels positively, and improvements have been noted in the free and total testosterone (TT) [12-18], indicating that excess body weight reduction can assist in restoring hormonal balance and enhancing individual metabolic health.

Nonetheless, there have been challenges regarding the best tool to use in assessing obesity status in individuals, particularly in relation to the body roundness index (BRI) and BMI. The cutoff value for obesity using BMI for adults is defined as a BMI of ≥ 30 kg/m², even as the cutoff value for BRI for adults is defined as a BRI of 4.00 and above for men and 5.00 and above for women. The BMI refers to the medical screening tool used in the measurement of the ratio of one’s height to his/her weight with the objective of approximating body fat levels. BMI is mainly computed by dividing the individual’s weight (in kilograms) by the square of his/her height (m^2^). BMI is commonly used as a key risk factor for the development and prevalence of numerous health issues and has been of immense importance in population-based studies owing to its wide approval in defining certain classes of body mass as health issues. Although BMI correlates to the amount of body fat in many individuals, a higher number is equated to more body fat; in some instances, it is inaccurate and fails to capture data regarding the fat mass in different parts of the body. As such, BMI should be used alongside other tests and tools in the assessment of the health and obesity status of individuals. Consequently, the BRI was developed in 2013 by mathematician Diana Thomas, who based her work on the idea that individuals are more similar to an egg than a cylinder and that one can utilize eccentricity in the measurement of how close an individual is to being circle-shaped [17-22]. Although BRI is considered a better indicator of health risks than BMI, it's unlikely to replace BMI in the near future for several reasons. For instance, regarding the aspect of ease of use, it is noteworthy that BMI is quick and easy to calculate while measuring body fat is more difficult, costly, and time-consuming [17]. Secondly, unlike BMI, in terms of accessibility, specialized tools and scales that measure body composition are not accessible to everyone, even as the BRI is still a newer technique that requires further validation [17,19-21]. Thirdly, BRI has been associated with the term “roundness,” which others might consider offensive and may be a key barrier to its future adoption and usage [17-19]. Regardless of these observations, BRI is still a better indicator of health risks, given that it accounts for fat distribution and muscle mass, which BMI does not [17]. Thus, BRI is calculated using height, hip size, and waist circumference and provides a value that typically varies between 1 and 20, with the lower and higher values indicating the health risk level [17-22]. In this regard, the BRI mainly measures the amount of belly fat (visceral fat) surrounding the inner organs in the abdomen, with higher amounts of belly fat being associated with increased risks for obesity-related diseases [17-22]. However, BRI may be calculated using an online calculator.

Regardless of the above observation, BMI is widely used to categorize individuals as underweight (below 18.5); healthy weight (between 18.5 and 25.0); overweight (between 25.0 and 30.0); obesity (30.0 and above); class 1 obesity (between 30.0 and 35.0); class 2 obesity (between 35.0 and 40.0); and class 3 obesity or severe obesity (40.0 and above), serving as a marker of body fat distribution. Higher BMI is strongly correlated with lower testosterone levels in men due to the increased conversion of testosterone to estrogen in adipose (fat) tissue, leading to a reduction in free and TT. This hormonal imbalance can exacerbate the symptoms of obesity, such as increased body fat, muscle weakness, fatigue, and reduced libido, creating a vicious cycle that worsens both physical and mental health [17-20]. Therefore, this review aims to synthesize the available evidence on the impact of WL on testosterone levels and hormonal health. By examining various WL strategies, including surgical, dietary, and lifestyle approaches, and their effects on testosterone levels, this article will provide a comprehensive understanding of how BMI and weight reduction influence hormonal health, with a focus on testosterone in men. Understanding these relationships is essential for developing effective strategies to combat obesity and its associated hormonal disorders, ultimately improving health outcomes for affected individuals.

## Review

Materials and methods

This study involved an in-depth literature search conducted on diverse virtual databases, including Scopus, PubMed, Google Scholar, Embase, and Web of Sciences, for studies and literature on the effects of WL on testosterone levels. The literature search utilized Boolean operators with every practicable combination of Medical Subject Headings (MeSH) terms, including testosterone, hypogonadism, obesity, WL, and insulin resistance. Moreover, the literature search strategy entailed two stages founded on Preferred Reporting Items for Systematic Reviews and Meta-Analyses (PRISMA) guidelines for the selection and inclusion of articles for systematic reviews and meta-analyses.

The initial phase entailed an independent screening of the titles and abstracts of the retrieved articles by two researchers. In case of sufficient data in the abstract to inform the decision to retain or exclude an article, the article exclusion decision was arrived at following full-text screening. Nevertheless, articles that have titles relevant to the systematic review but with inadequate abstract data were further included to undergo the second phase, which involved full-text screening. The final phase entailed the full-text screening of all retained articles for either inclusion or exclusion, based on the set criteria. Potential disputes were mainly resolved through a third researcher tasked with deciding on the article’s suitability for inclusion or exclusion, with the decision being arrived at via consensus and consultations.

Study inclusion and exclusion criteria

For this systematic review, the study inclusion criteria involved original studies, such as crossover design studies, prospective cohort studies, and randomized controlled trials that met the following criteria: studies on the effects of WL on testosterone levels, studies published in the English language, and studies published in the last 15 years. Further, the studies excluded included those that were sponsored by clinical trials, editorials, and opinion pieces. The abstract evaluation resulted in the removal of 350 articles. Additionally, the important data extraction from identified and included qualified studies was conducted based on the following factors: (a) the study’s general characteristics, such as the authors’ names, study year, publication year, and sampling methods utilized; (b) the study population characteristics, including age, race, sample size, gender, and follow-up; (c) intervention type and duration; as well as (d) the main study findings.

Data collection process

Data extraction from the included studies was under the supervision of the authors. Potential discrepancies and disputes were mainly solved through the use of consultations and discussions. Further, the information regarding the authors, publication year, study location, study sample size, response rate, and screening tool employed was independently extracted from every study by the author using a standardized data extraction format.

Quality assessment

The included studies' quality was assessed using the Grading of Recommendations, Assessment, Development, and Evaluations (GRADE) approach [20]. Though the GRADE approach implies a systematic framework used to evaluate evidence and recommend healthcare guidelines, it is mainly used in systematic review studies to assess evidence quality and the strengths of the recommendations [20]. Thus, the GRADE method assesses the certainty of evidence across studies based on factors such as study design, risk of bias, inconsistency, indirectness, imprecision, and other considerations, providing a systematic framework to determine the strength of evidence for each outcome [20]. The evidence assessment process entails the definition of the question, study population, alternatives, and important outcomes by the authors, followed by rating each outcome’s evidence quality through a scale comprising very low, low, moderate, and high [20]. To establish the strength of a recommendation, the researchers have to consider the evidence quality, preferences and values, the balance between the desirable and undesirable effects, as well as resource considerations. The GRADE approach aims to enhance the consistency, transparency, and rigor of evidence assessment in systematic reviews.

Results

**Figure 1:**
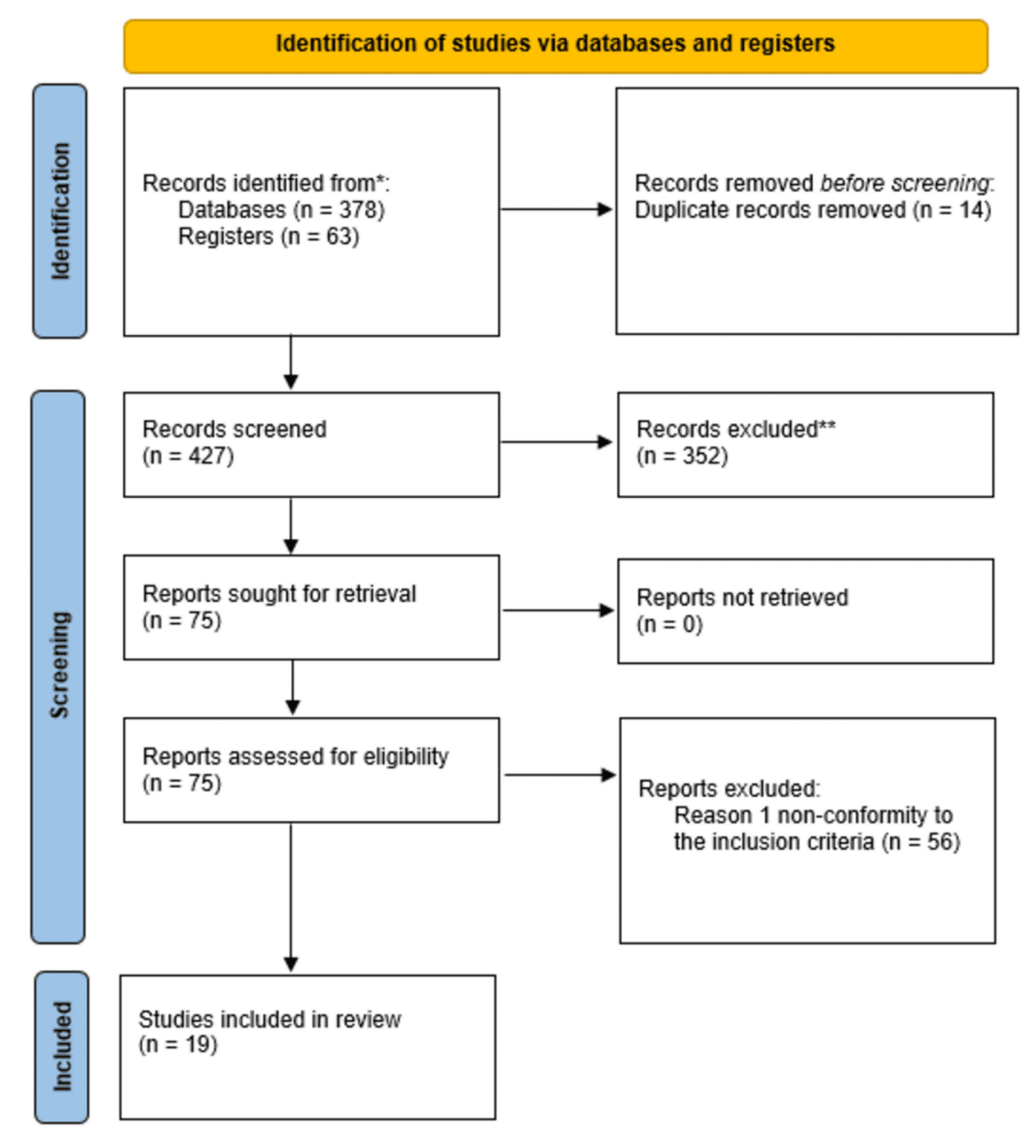
PRISMA flowchart for included studies PRISMA: Preferred Reporting Items for Systematic Reviews and Meta-Analyses *: included from the database; **: excluded from the database

A total of 441 studies were identified through an in-depth search of various online databases and other sources. Following the removal of 14 duplicates, a total of 427 studies were subjected to screening of titles and abstracts, leading to the removal of 352 records owing to their irrelevance to the research topic. Subsequently, a full-text screening of the remaining 75 articles was conducted leading to the exclusion of 56 articles for failure to conform to set study inclusion criteria. As a result, a total of 19 studies met the inclusion criteria and were for to be eligible for inclusion in this systematic review. Figure 1 below provides a detailed overview of the literature search and selection process conducted using PRISMA.

The studies explored various methods of WL, including bariatric surgery, dietary interventions, and lifestyle changes, and their effects on sex hormone levels, semen parameters, and fertility outcomes. The following section provides a detailed analysis of the key findings from each study, categorizing them into specific themes based on the type of intervention and its observed effects on reproductive health. Furthermore, the results from the studies summarized in this review reveal significant insights into the impact of WL on reproductive health and hormonal balance in both men and women with obesity. A PRISMA flowchart was created to summarize the selection process of the eligible studies. Potential content was extracted from selected studies. The summary of the findings of the studies included in this systematic review has been presented in Table 1 below.

**Table 1:** The relationship between weight loss and hormonal changes in obese men: findings from recent research GRADE: Grading of Recommendations, Assessment, Development, and Evaluations; PSA: prostate-specific antigen; SHBG: sex hormone-binding globulin; LH: luteinizing hormone; FSH: follicle-stimulating hormone; TT: total testosterone; AE: androstenedione; E2: estradiol; DHEA: dehydroepiandrosterone; CR: calorie restriction; TRE: time-restricted eating; RYGB: Roux-en-Y gastric bypass; OAGB: one-anastomosis gastric bypass; HR-QOL: health-related quality of life; AI: aromatase inhibitor; BMI: body mass index

First author name/citation	Year of publication	Country	Study design	Aim of study	Sample size	Study outcome	Key findings	Conclusion	GRADE rating
Di Vincenzo et al. [21]	2018	Italy	Prospective observational study	To investigate the impact of short-term weight loss from sleeve gastrectomy on plasma levels of sex steroids and PSA concentration in men with severe obesity.	29 obese men, 19 lean controls	Impact of sleeve gastrectomy on sex steroids and PSA levels	Sleeve gastrectomy significantly increased total testosterone and PSA levels, and reduced estradiol levels in obese men with hypogonadism.	Sleeve gastrectomy reverses hypogonadism rapidly post-surgery and may increase PSA, needing long-term monitoring for prostate health.	High
Wood et al. [22]	2020	Brazil	Prospective study	To evaluate the effects of bariatric surgery on reproductive hormone levels, semen analysis, and sperm DNA fragmentation in men with severe obesity.	42 obese men, 32 fertile controls	Impact of bariatric surgery on reproductive hormones, semen, and DNA fragmentation	Bariatric surgery improves testosterone and sperm DNA fragmentation but worsens sperm concentration post-surgery.	Bariatric surgery enhances reproductive hormone levels but reduces sperm concentration, requiring careful monitoring.	High
Jedamzik et al. [23]	2023	Austria	Retrospective analysis	To evaluate the impact of OAGB and RYGB on testosterone levels and weight loss in male patients with obesity.	Not specified	Changes in testosterone levels and weight loss	Significant increase in testosterone levels post-surgery; changes correlated with percent excess weight loss; no difference between surgical methods.	Serum testosterone levels significantly rise after metabolic and bariatric surgery, playing a role in continued weight loss irrespective of the surgical method used.	Moderate
Ezzat et al. [24]	2021	Not specified	Cohort study	To evaluate the consequences of weight reduction by bariatric surgery on androgen levels and ovarian volume in women with PCOS.	36	Changes in androgen levels and ovarian volume	Significant reduction in body mass index, and testosterone levels; increase in SHBG and regulation of menstrual cycle; decreased ovarian volume post-surgery.	Bariatric surgery results in durable weight loss and restores normal physiological androgen levels and ovarian morphology in infertile women with PCOS.	High
Emami et al. [25]	2021	Iran	Systematic review and meta-analysis	To systematically review and analyze the effects of bariatric surgery on endogenous sex hormone levels and sex hormone-binding globulin levels.	N/A	Impact of bariatric surgery on sex hormones	Bariatric surgery caused significant changes in sex hormones, increasing LH, FSH, TT, SHBG, and reducing AE, E2, and DHEA in both males and females. No significant changes were observed in progesterone or prolactin.	Bariatric surgery significantly affects sex hormone levels, improving hormonal balance and potentially sexual function. Larger trials are needed for more specific conclusions.	High
Whittaker et al. [26]	2021	Various	Systematic review and meta-analysis	To assess the relationship between low-fat diets and testosterone levels in men through a systematic review and meta-analysis of existing intervention studies.	206	Effects of low-fat diets on testosterone levels in men	Low-fat diets significantly reduced total, free, and urinary testosterone levels. Men of European ancestry experienced a greater decrease in testosterone with low-fat diets.	Low-fat diets decrease testosterone levels in men. Further randomized controlled trials are necessary to confirm these findings, particularly among different ethnic groups.	High
Whittaker et al. [27]	2022	Not specified	Systematic review and meta-analysis	To investigate the effects of low-carbohydrate diets on men's cortisol and testosterone levels.	309	Comparison of low- versus high-carbohydrate diets	Short-term low-carb diets increase resting cortisol; long-term diets have no consistent effect on resting cortisol; high-protein low-carb diets decrease resting total testosterone.	Low-carbohydrate diets affect cortisol levels initially, while high-protein diets may lead to a significant decrease in resting testosterone levels.	High
Ma et al. [28]	2024	USA	Observational, data analysis from NHANES	To investigate the correlation between percent body fat and testosterone levels in men.	5959	Negative correlation between testosterone and body fat	Testosterone levels were negatively correlated with total body fat, android fat, gynoid fat, and android/gynoid ratio in men. In women, no significant correlation between testosterone and body fat was observed.	Testosterone levels are inversely related to body fat percentages in men, reinforcing the negative association between obesity and testosterone. This correlation was not significant in women.	High
Santi et al. [29]	2024	Not specified	Meta-analysis	To examine the influence of body weight loss on semen parameters in obese men.	345	Semen analysis parameters before and after weight loss	Significant increase in sperm concentration and progressive motility; significant decrease in sperm DNA fragmentation index after weight loss.	Body weight loss may improve qualitative and quantitative sperm characteristics, suggesting weight loss for male partners with obesity and semen analysis alterations in couples attempting conception.	High
Salas-Huetos et al. [30]	2021	Spain	Systematic review and meta-analysis	To review and analyze the relationships between male adiposity, sperm parameters, and reproductive hormone levels.	169 studies, 60 qualitative, 28 quantitative	Impact of adiposity on sperm quality and reproductive hormones	Overweight/obesity linked to lower sperm quality (volume, count, concentration, motility) and higher estradiol levels.	Maintaining healthy body weight improves sperm quality and fertility parameters.	High
Kyinn et al. [31]	2021	USA	Longitudinal Study	To investigate the rates of weight gain and obesity among transgender and gender-diverse individuals before and during hormone therapy.	470	Weight gain and obesity rates before and during hormone therapy	Transmasculine individuals gained more weight and had higher obesity rates compared to transfeminine individuals during gender-affirming hormone therapy. Weight gain occurred earlier and was more significant in transmasculine.	Transmasculine individuals have higher rates of obesity and weight gain. Regular monitoring and weight-reduction interventions are recommended during hormone therapy.	Moderate
Lin et al. [32]	2024	USA	Randomized controlled trial (RCT)	To compare the effects of TRE and daily calorie restriction on sex hormone levels in males and females with obesity.	90	TRE vs daily calorie restriction on sex hormones	Both TRE and CR led to significant weight loss, but there were no changes in sex hormone levels (testosterone, DHEA, SHBG) in males, premenopausal females, or postmenopausal females. Estradiol, estrone, and progesterone also remained unchanged in postmenopausal females.	TRE and CR produce significant weight loss in males and females with obesity, but neither has an effect on circulating sex hormone levels over 12 months.	High
Ikehata et al. [33]	2023	Not specified	Mendelian Randomization	To investigate potential causal associations between body composition and testosterone levels.	Not specified	Causal associations between body composition and testosterone levels	Genetically predicted whole body fat mass negatively associated with total testosterone (TT), bioavailable testosterone (BT), and SHBG. The effect of fat mass was more potent than fat-free mass.	Reducing fat mass may increase testosterone levels.	Moderate
Giagulli et al. [34]	2020	Italy	Retrospective cohort study	To assess whether weight loss has a greater effect on improving testosterone levels compared to glycemic control in obese men with type 2 diabetes and hypogonadism.	71 obese men with T2DM	Weight loss vs glycemic control on testosterone levels in diabetic men	94% of patients achieving >10% weight loss showed increased testosterone, while glycemic control did not improve testosterone levels.	Weight loss has a greater impact on testosterone levels in obese T2DM men than glycemic control.	High
Pasquali et al. [35]	2020	Europe	Clinical guideline	To provide guidelines for the endocrine evaluation and management of obesity-related conditions.	N/A	Endocrine work-up in obesity	Hormonal imbalances (e.g., hypogonadism) are prevalent in obesity and must be addressed during endocrine work-ups.	Weight loss is crucial to restore hormonal balance; endocrine disorders modestly affect weight loss.	High
Mayneris-Perxachs et al. [36]	2020	Spain	Observational, longitudinal study	To investigate the influence of gut microbiota on gonadal steroid levels and the implications of sexual dimorphism, obesity, and menopausal status.	Not specified	Gut microbiota differences and gonadal steroid correlations	Sexual dimorphism in gut microbiota was eliminated by menopausal and obesity status. Gut microbiota differences were linked with testosterone and progesterone levels. Microbial signatures could predict testosterone levels.	Gut microbiota composition and functionality differ between men and women but are impacted by obesity and menopausal status. Microbiome composition was linked to circulating gonadal steroids, especially testosterone, with obesity diminishing these differences.	Moderate
Grossmann et al. [37]	2024	Australia	Randomized controlled trial (RCT)	To evaluate the effects of testosterone treatment combined with weight loss on health-related quality of life and psychosocial function in men.	1007	Testosterone therapy effects on HR-QOL and weight loss	Testosterone treatment improved social status and sense of coherence but had minimal impact on HR-QOL overall. Weight loss from lifestyle interventions had a greater positive impact on HR-QOL, particularly for those with better physical function and fewer depressive symptoms at baseline.	Weight loss through lifestyle changes, rather than testosterone treatment, had a more significant effect on health-related quality of life. Testosterone therapy mainly impacted subjective social status and coherence.	High
Colleluori et al. [38]	2020	Not specified	Randomized double-blind placebo-controlled pilot trial	To evaluate the efficacy and safety of weight loss (WL) plus aromatase inhibitor (AI) therapy in severely obese men with hypogonadotropic hypogonadism (HHG).	23 obese men (BMI≥35 kg/m²), 35-65 years old	Improvement in hormonal profile with AI+WL compared to WL alone	AI+WL group showed higher testosterone (p=0.003) and lower estradiol (p=0.001); no significant differences in muscle strength or hypogonadism symptoms; AI+WL led to greater fat loss without affecting lean mass, and better bone stability compared to placebo.	AI+WL is effective in reversing the hormonal profile of HHG in severely obese men but does not provide additional benefits in muscle strength or hypogonadism symptoms beyond WL alone.	High
Mangolim et al. [39]	2018	Not specified	Systematic review of randomized controlled trials	To analyze the effectiveness of testosterone therapy for weight loss, controlling obesity complications, and preventing cardiovascular events in obese men with low testosterone levels.	Not applicable (protocol for review)	Expected outcomes include weight loss, adverse events, quality of life, libido improvement, control of obesity complications, and cardiovascular event frequency	Preliminary evidence suggests inconsistent results regarding the benefits of testosterone therapy in obese men with low testosterone levels.	The systematic review aims to assess the safety and efficacy of testosterone therapy in obese men for weight loss, quality of life improvements, and cardiovascular event prevention compared to placebo or no treatment.	Moderate

Discussion

Impact of Bariatric Surgery on Sex Hormones and Reproductive Health

Bariatric surgery has been increasingly recognized for its profound effects on sex hormones and reproductive health in individuals with obesity. Several studies have explored these impacts, particularly in men with obesity-related hypogonadism. Di Vincenzo et al. conducted a prospective observational study on 29 obese men with hypogonadism and 19 lean controls to assess the effects of short-term WL following sleeve gastrectomy [21]. The study revealed a significant increase in TT levels and prostate-specific antigen (PSA) concentrations, alongside a reduction in estradiol levels post-surgery [21]. These findings suggest that sleeve gastrectomy rapidly reverses hypogonadism by reducing fat mass, which in turn lowers aromatase activity and thus decreases the conversion of testosterone into estrogen. The increase in PSA levels, while indicating improved androgen status, also signals the importance of long-term prostate health monitoring in these patients, as elevated PSA levels can be associated with an increased risk of prostate issues [21]. Also, Wood et al. conducted a similar study on 42 obese men and 32 fertile controls, focusing on reproductive hormones, semen analysis, and sperm DNA fragmentation [22]. They found that bariatric surgery significantly improved testosterone levels and reduced sperm DNA fragmentation, which is essential for better reproductive outcomes. However, the study also noted a reduction in sperm concentration post-surgery, suggesting that while hormonal balance improves, there may be trade-offs in sperm production that require careful reproductive health monitoring after surgery [22].

In addition to these studies in men, research has also examined the effects of different types of bariatric surgery. Jedamzik et al. performed a retrospective analysis of the effects of one-anastomosis gastric bypass (OAGB) and Roux-en-Y gastric bypass (RYGB) on testosterone levels and WL in male patients with obesity [23]. Their findings showed significant increases in testosterone levels following both surgical procedures, with these hormonal changes closely correlated with the percentage of excess weight lost [23]. Interestingly, the study found no difference in the impact on testosterone levels between OAGB and RYGB, suggesting that both procedures are equally effective in restoring hormonal balance and promoting WL in men with obesity [23]. This aligns with previous research showing that fat reduction, regardless of the specific surgical method, plays a critical role in restoring testosterone levels by reducing the amount of aromatase enzyme, which is responsible for converting testosterone into estrogen in adipose tissue [20-23]. These findings underscore the importance of weight reduction in addressing hormonal imbalances in obese men and the potential benefits of bariatric surgery in improving overall metabolic and reproductive health [23].

Bariatric surgery has also been shown to benefit women, particularly those with polycystic ovary syndrome (PCOS), a condition commonly associated with obesity and hormonal imbalances. Ezzat et al. conducted a cohort study on 36 women with PCOS to assess the effects of bariatric surgery on androgen levels, ovarian volume, and menstrual regulation [24]. The study found significant reductions in BMI and testosterone levels, as well as an increase in SHBG post-surgery [24]. These hormonal changes were accompanied by a regulation of the menstrual cycle and a decrease in ovarian volume, suggesting that bariatric surgery can restore normal physiological androgen levels and ovarian morphology in women with PCOS. Additionally, Emami et al. conducted a systematic review and meta-analysis that examined the broader effects of bariatric surgery on sex hormones in both men and women [25]. The findings of the study have confirmed that bariatric surgery leads to significant improvements in hormonal profiles, including increases in LH, follicle-stimulating hormone (FSH), TT, and SHBG, alongside reductions in androstenedione (AE), estradiol, and dehydroepiandrosterone (DHEA) [25]. While these changes support the use of bariatric surgery as a treatment for endocrine dysfunction, the long-term effects on reproductive health warrant further investigation.

Weight Loss Through Dietary Interventions and Hormonal Balance

Dietary interventions, particularly low-fat and low-carbohydrate diets, have been widely studied for their impact on WL and reproductive hormones, especially testosterone and cortisol, which are crucial for male reproductive health. Whittaker and Wu conducted a systematic review and meta-analysis involving 206 men to examine the effects of low-fat diets on testosterone levels [26]. The findings indicated that low-fat diets led to significant reductions in total, free, and urinary testosterone levels, with men of European ancestry showing a greater decline compared to other ethnic groups. While low-fat diets are often effective for WL, these results raise concerns about their potential negative impact on male reproductive health by lowering testosterone levels [26]. The study underscores the need for further randomized controlled trials to verify these effects across different ethnic populations and to understand the mechanisms behind these hormonal changes.

Still, a complex relationship has been noted between glucagon-like peptide-1 (GLP-1), sex hormones, and glucose-dependent insulinotropic polypeptide (GIP), even as the effects diverge on the basis of condition and gender [1-5,14-20]. For instance, in healthy men, acute GLP-1 administration has no significant impact on testosterone levels. Consequently, in obese men, chronic treatment using GLP-1 receptor agonists (GLP-1 RAs) has been found to improve reproductive hormone levels, particularly in obese males with type 2 diabetes and hypogonadism [1-5,14-20,26]. This has been attributed to the WL linked to GLP-1 RAs. Consequently, studies have disclosed that, in female rats, the central administration of GIP reduces the circulating FSH levels [2,5,26-28]. Still, studies conducted on mice have revealed that GIPR and GLP-1R mice had longer estrous cycles, reduced fertility, and fewer pups produced [1,2,5,26-28]. Thus, the GLP-1 and GIP are incretin hormones secreted in the intestine following consumption of glucose or various nutrients and are known to stimulate insulin secretion from pancreatic β cells [1,2,5,26]. The increased abundance of GLP-1 receptors within the reproductive system is indicative of the correlations between incretins and the sex and fertility hormones [1,2,5,26]. Nevertheless, further studies are required to confirm GLP-1 RAs’ potential role in the treatment of male infertility.

In another study, Whittaker and Harris explored the effects of low-carbohydrate diets on cortisol and testosterone levels [27]. Their systematic review and meta-analysis revealed that short-term low-carb diets increased resting cortisol levels, though these changes normalized over time [27]. Long-term low-carbohydrate diets had no consistent impact on cortisol, but high-protein, low-carbohydrate diets were associated with a significant reduction in resting testosterone levels [27]. These results suggest that macronutrient composition, particularly the balance of carbohydrates and protein, plays a critical role in hormonal regulation. Furthermore, Ma et al. analyzed data from 5,959 men, finding a negative correlation between body fat percentage - especially abdominal fat - and testosterone levels [28]. This supports the view that higher body fat, particularly in the android region, is inversely related to testosterone, contributing to hormonal imbalances in obese individuals. These studies highlight the complex relationship between diet, fat distribution, and hormonal health, particularly in men.

Consequently, the relationship between obesity and low testosterone is multifactorial, involving hormonal, metabolic, and inflammatory pathways. For instance, obesity increases aromatase activity in adipose tissue through the aromatase conversion of testosterone to estrogen [1,2,5]. Thus, the effect includes the observation that, in obese individuals, the higher amount of adipose tissue increases aromatase activity, leading to greater conversion of testosterone to estrogen [1,2,5]. Elevated estrogen levels can suppress the HPG axis, reducing gonadotropin-releasing hormone (GnRH) and LH secretion, which further lowers testosterone production. Also, obesity results in the suppression of the HPG axis, which is a critical endocrine pathway tasked with the regulation of reproductive function through the interaction between the hypothalamus, pituitary gland, and gonads [3,14]. Thus, an increment in estrogen and inflammatory markers in obesity leads to the disruption of the HPG axis. This results in reduced GnRH secretion by the hypothalamus, which in turn decreases LH and FSH release from the pituitary gland [2,5,19,20]. These changes lead to reduced stimulation of the testes and decreased testosterone production.

Still, obesity has been acknowledged to result in insulin resistance, leading to compensatory hyperinsulinemia (high insulin levels). Thus, chronic hyperinsulinemia may inhibit testosterone production by affecting LH release and by direct effects on testicular function. Insulin resistance can also exacerbate adiposity, creating a feedback loop that further suppresses testosterone [4,20-26]. Obesity has also been associated with increased levels of inflammatory cytokines, including TNF-alpha, IL-6, and oxidative stress [8-12,23]. Thus, the inflammatory cytokines interfere with Leydig cell function (cells in the testes that produce testosterone) and can impair LH stimulation of testosterone production. This inflammation also affects the HPG axis, suppressing testosterone levels.

Further, leptin, a hormone produced by adipose tissue, is often elevated in obesity but with reduced sensitivity at the hypothalamus due to leptin resistance. As such, leptin resistance impairs the normal stimulatory effects of leptin on the HPG axis. While leptin generally promotes testosterone synthesis, in obesity, high leptin levels do not lead to increased testosterone due to leptin resistance and dysregulated signaling [1,2,19,20,22-26]. Additionally, obesity has been acknowledged as a key risk factor for obstructive sleep apnea, which disrupts sleep quality and reduces rapid eye movement (REM) sleep. Poor sleep and sleep apnea reduce the nocturnal pulses of testosterone release, further lowering overall testosterone levels. Sleep quality has a direct influence on testosterone production, as REM sleep is essential for optimal HPG axis function [16,19-25]. Lastly, adipokines, which refer to the hormones produced by adipose tissue, including resistin and adiponectin, are often imbalanced in obesity. The low adiponectin and high resistin levels contribute to insulin resistance, inflammation, and further impairment of the HPG axis, thereby exacerbating low testosterone levels [11].

Effects of Weight Loss on Male Fertility Parameters

WL has been consistently associated with improvements in semen parameters and male fertility outcomes. Studies have shown that reducing body fat can lead to enhanced sperm quality, concentration, and motility, as well as reductions in sperm DNA fragmentation, which are critical for reproductive success. Additionally, Santi et al. conducted a meta-analysis on 345 obese men to examine the influence of body WL on semen parameters [29]. The analysis revealed significant improvements in sperm concentration and progressive motility, along with a reduction in sperm DNA fragmentation index following WL [29]. These findings indicate that WL not only improves the qualitative and quantitative aspects of sperm but also enhances the overall fertility potential in obese men. The study suggests that WL interventions should be considered in obese men experiencing altered semen parameters, particularly in couples attempting conception.

Furthermore, in a systematic review and meta-analysis, Salas-Huetos et al. examined the relationship between male adiposity, sperm parameters, and reproductive hormones across 169 studies [30]. The analysis revealed that overweight and obesity were linked to lower sperm quality, including reductions in sperm volume, count, concentration, and motility. Additionally, higher estradiol levels were observed in obese men, further contributing to the disruption of reproductive hormone balance [30]. These findings highlight the importance of maintaining a healthy body weight to improve sperm quality and fertility outcomes in men.

Weight Gain and Hormonal Changes in Transgender Individuals

Weight gain and obesity are common concerns during gender-affirming hormone therapy in transgender and gender-diverse individuals. Hormone therapy often leads to changes in body composition, which can exacerbate obesity-related conditions and further impact hormonal balance. Kyinn et al. conducted a longitudinal study on 470 transgender and gender-diverse individuals to investigate the rates of weight gain and obesity before and during hormone therapy [31]. The study found that transmasculine individuals gained more weight and had higher rates of obesity compared to transfeminine individuals during gender-affirming hormone therapy [31]. Weight gain occurred earlier and was more significant in transmasculine individuals, suggesting that this population may be at a higher risk for obesity-related complications during hormone therapy [31]. Regular weight monitoring and weight-reduction interventions are recommended to mitigate the risks associated with hormone therapy in transgender individuals.

Comparison of Time-Restricted Eating and Daily Calorie Restriction

In a randomized controlled trial by Lin et al., the effects of time-restricted eating (TRE) and daily calorie restriction on sex hormone levels were compared in 90 obese males and females [32]. Both interventions led to significant WL, but there were no observed changes in sex hormone levels, including testosterone, DHEA, and SHBG, in males or premenopausal and postmenopausal females. Estradiol, estrone, and progesterone levels also remained unchanged in postmenopausal females [32]. These findings suggest that while both TRE and CR are effective weight-loss strategies, they may not significantly impact circulating sex hormone levels over 12 months in obese individuals.

Influence of Fat Mass on Total and Bioavailable Testosterone

**Figure 2:**
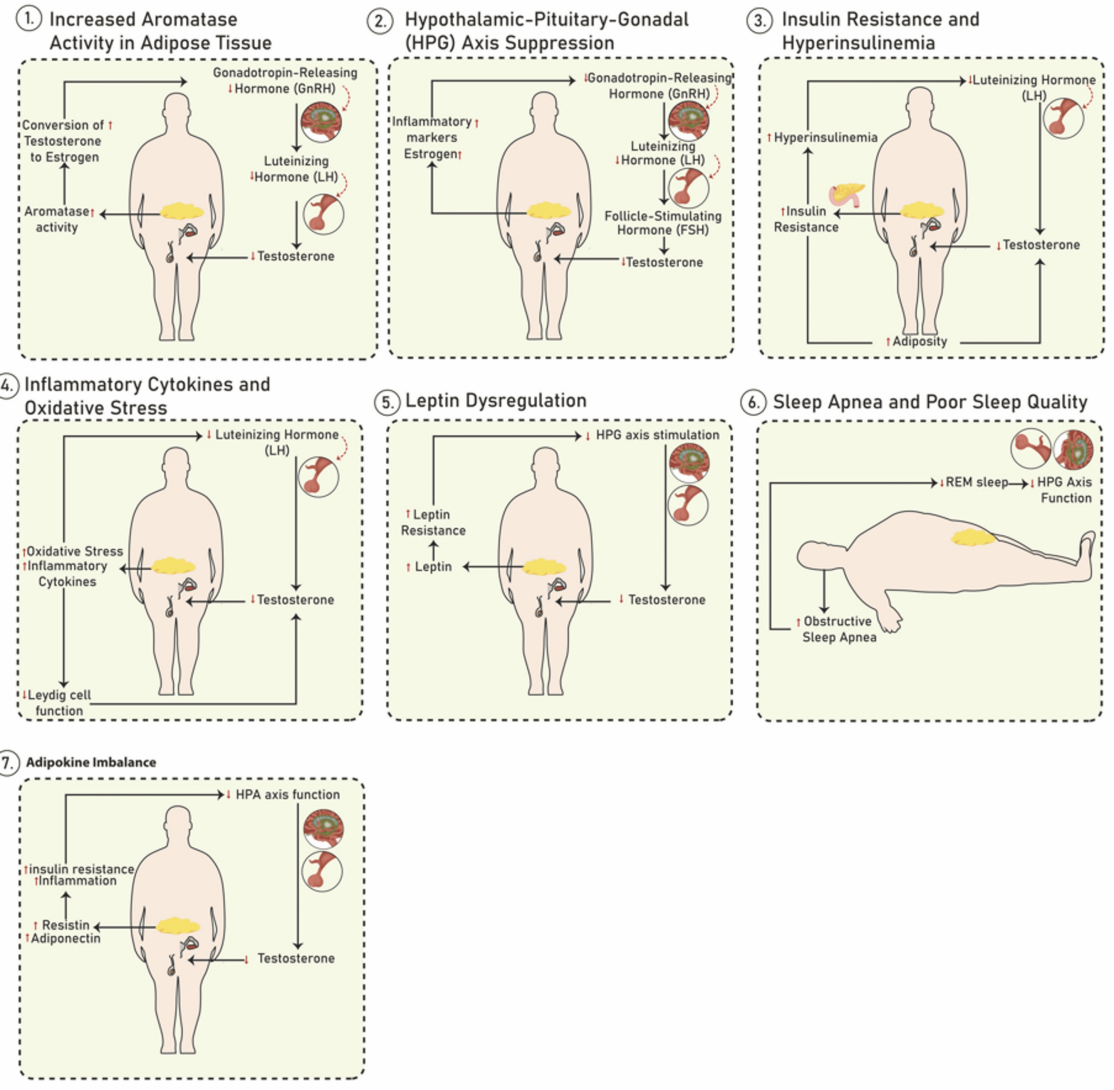
The relationship between obesity and low testosterone Image Credits: Okelue E. Okobi

In the Ikehata et al. study, Mendelian randomization was employed to explore causal relationships between body composition and testosterone levels [33]. The analysis revealed that genetically predicted whole body fat mass was negatively associated with TT, bioavailable testosterone (BT), and SHBG. Specifically, the estimates indicated a significant reduction in TT and BT levels with increasing fat mass. Additionally, the results demonstrated that the detrimental effect of fat mass on testosterone levels was more pronounced than that of fat-free mass [33]. These findings highlight the importance of reducing fat mass to enhance testosterone levels potentially. The overall effect of obesity and testosterone is exemplified in Figure 2.

Weight Loss as a Key Modulator of Testosterone and Hormonal Balance in Obesity

Giagulli et al. showed that WL, especially greater than 10%, significantly improved testosterone levels in obese men with type 2 diabetes (T2DM), outperforming glycemic control alone. Pasquali et al. emphasized that WL is key to restoring hormonal balance in obese individuals, particularly addressing hypogonadism, as highlighted in clinical guidelines for obesity management [34,35]. Mayneris-Perxachs et al. introduced the role of gut microbiota in regulating gonadal steroids, demonstrating that obesity alters typical sex-based differences in gut microbiota composition, impacting testosterone levels. Their findings suggested that microbial signatures could predict testosterone levels, offering a novel dimension to the interaction between obesity and hormonal health [36]. Grossmann et al. evaluated the effects of testosterone therapy and WL on health-related quality of life (HR-QOL) [37]. The study found that while testosterone therapy improved psychosocial factors like social status, WL through lifestyle interventions had a more significant positive impact on HR-QOL, particularly for those with better physical function and fewer depressive symptoms at baseline [37].

Overall, WL, whether through lifestyle changes or bariatric surgery, was identified as the primary intervention for improving testosterone levels and hormonal health in obese men. While testosterone therapy provided modest psychosocial benefits, the most substantial improvements in HR-QOL and hormonal balance were achieved through significant reductions in BMI. The role of gut microbiota also emerged as a potential area for future interventions to enhance testosterone regulation in obese populations.

Comparative Outcomes of Aromatase Inhibitors and Testosterone Therapy in Obesity-Related Hypogonadism

The studies by Colleluori et al. and Mangolim et al. addressed the role of testosterone therapy and WL interventions in obese men with hypogonadism and low testosterone levels [38,39]. Colleluori’s randomized controlled trial demonstrated that a combination of aromatase inhibitor (AI) therapy and WL significantly improved the hormonal profile of obese men with hypogonadotropic hypogonadism (HHG) [38]. Specifically, the AI+WL group showed a significant increase in testosterone levels and a reduction in estradiol compared to the placebo group (p=0.003 and p=0.001, respectively). However, no substantial improvement was observed in muscle strength or hypogonadism symptoms, although AI+WL did result in more fat loss without affecting lean mass and better bone stability compared to placebo. Consequently, Mangolim et al. proposed a systematic review protocol to evaluate testosterone therapy's effectiveness for WL and the prevention of obesity-related complications, including cardiovascular events [39]. The preliminary data indicate inconsistent results, with some studies suggesting minimal benefits of testosterone therapy in this population. The review aims to further clarify the therapy's role in promoting WL, improving quality of life, controlling complications, and reducing cardiovascular event frequency in obese men with low testosterone levels.

Key findings from the literature

Bariatric surgery consistently improves testosterone levels in men and reduces androgen levels in women with PCOS, leading to improved reproductive health outcomes. However, surgery may reduce sperm concentration in men, necessitating careful reproductive monitoring. Low-fat and low-carbohydrate diets, though effective for WL, can negatively impact testosterone levels, with high-protein, low-carb diets particularly reducing resting testosterone. WL significantly improves semen parameters, including sperm concentration, motility, and DNA fragmentation, in obese men, enhancing fertility outcomes. Transgender individuals, especially transmasculine, are at higher risk for obesity-related complications during hormone therapy and require regular weight monitoring. TRE and daily calorie restriction, while effective for WL, may not significantly impact sex hormone levels in obese individuals over a 12-month period. These results collectively highlight the complex interplay between WL, reproductive health, and hormonal balance in obese individuals. Further research is required to explore the long-term effects of various WL interventions on fertility and endocrine function across diverse populations.

Strengths and limitations

The review article on hormonal health and obesity presents several strengths that enhance its contributions to the field. It offers a comprehensive synthesis of recent research, integrating findings from observational studies, randomized controlled trials, and meta-analyses to provide a well-rounded understanding of how BMI influences hormonal health. By including various interventions - such as bariatric surgery, dietary modifications, and hormonal therapies - the article facilitates a multifaceted exploration of the topic, relevant for both clinical practice and future research. It also emphasizes individual variability in responses to WL, considering factors like age, ethnicity, and comorbidities, adding depth to the discussion and identifying gaps in the literature that warrant further investigation.

However, the review has notable limitations. Variability in study designs, sample sizes, and methodologies can introduce bias and affect the reliability of conclusions. Conflicting outcomes due to differing participant demographics may limit the generalizability of the findings. Additionally, some studies lacked long-term follow-up data, making it challenging to assess the sustained effects of WL on testosterone levels. While the article calls for further research, it does not provide specific recommendations for study designs that could enhance future investigations. These limitations should be considered when interpreting the review's findings and implications for clinical practice.

## Conclusions

This review highlights the significant impact of WL on hormonal balance and reproductive health in individuals with obesity, with a focus on both men and women. Bariatric surgery consistently improves testosterone levels and restores hormonal balance, particularly in men with obesity-induced hypogonadism and women with PCOS. However, bariatric surgery may reduce sperm concentration, warranting close post-surgical reproductive monitoring. In contrast, dietary interventions such as low-fat and low-carbohydrate diets, while effective for WL, can negatively affect testosterone levels, particularly in men following high-protein, low-carb regimens. WL also leads to improvements in sperm quality, including enhanced sperm concentration, motility, and reduced DNA fragmentation, thereby improving fertility outcomes. Moreover, the results from studies on transgender individuals undergoing hormone therapy reveal a higher risk of weight gain and obesity, especially in transmasculine individuals, underlining the need for tailored weight management strategies. While TRE and daily calorie restriction successfully promote WL, neither was found to significantly alter sex hormone levels over a 12-month period. Overall, WL interventions, particularly bariatric surgery, are effective in improving reproductive health, but careful attention must be paid to potential adverse effects on fertility parameters. Future research should focus on long-term outcomes and optimizing WL strategies to maintain hormonal balance and fertility in diverse populations.
